# Computational Prediction of Human Salivary Proteins from Blood Circulation and Application to Diagnostic Biomarker Identification

**DOI:** 10.1371/journal.pone.0080211

**Published:** 2013-11-12

**Authors:** Jiaxin Wang, Yanchun Liang, Yan Wang, Juan Cui, Ming Liu, Wei Du, Ying Xu

**Affiliations:** 1 Key Laboratory for Symbolic Computation and Knowledge Engineering of the Ministry of Education, College of Computer Science and Technology, Jilin University, Changchun, China; 2 Department of Biochemistry and Molecular Biology, and Institute of Bioinformatics, University of Georgia, Athens, Georgia, United States of America; Chinese Academy of Sciences, China

## Abstract

Proteins can move from blood circulation into salivary glands through active transportation, passive diffusion or ultrafiltration, some of which are then released into saliva and hence can potentially serve as biomarkers for diseases if accurately identified. We present a novel computational method for predicting salivary proteins that come from circulation. The basis for the prediction is a set of physiochemical and sequence features we found to be discerning between human proteins known to be movable from circulation to saliva and proteins deemed to be not in saliva. A classifier was trained based on these features using a support-vector machine to predict protein secretion into saliva. The classifier achieved 88.56% average recall and 90.76% average precision in 10-fold cross-validation on the training data, indicating that the selected features are informative. Considering the possibility that our negative training data may not be highly reliable (i.e., proteins predicted to be not in saliva), we have also trained a ranking method, aiming to rank the known salivary proteins from circulation as the highest among the proteins in the general background, based on the same features. This prediction capability can be used to predict potential biomarker proteins for specific human diseases when coupled with the information of differentially expressed proteins in diseased *versus* healthy control tissues and a prediction capability for blood-secretory proteins. Using such integrated information, we predicted 31 candidate biomarker proteins in saliva for breast cancer.

## Introduction

Human blood has long been used as an information source for detection of human diseases such as liver enzymes for detecting hepatitis, white-blood cell counts for infection detection and prostate-specific antigen (PSA) for diagnosing prostate cancer. In comparison, human saliva has not been used for the same purposes nearly as much. Recent large-scale proteomic analyses have revealed that human saliva is also rich in proteins [1], some of which come from the blood circulation and hence can potentially serve as a general information pool for disease biomarker identification. This study is on the development of a computational method for identification of the distinct features of salivary proteins that come from circulation and an application of the identified features to predict proteins that can get into saliva from circulation. 

The earliest work on using salivary proteins as disease biomarkers of distal organs can be traced back to 1986 when the Kallikreina salivary biomarkers for detection of breast cancer and gastrointestinal cancer were published [2]. Since then, a number of salivary proteins have been found to have elevated levels in patients of specific cancer types compared to the healthy population such as PSA for prostate cancer [3], c-tumor protein erbB-2 and p53 for breast cancer [4]. While a few salivary proteins have been found to be relevant to specific diseases, there has not been a general and effective approach for identifying disease markers in saliva, to the best of our knowledge. 

The current understanding about how biomolecules can move from circulation into saliva can be summarized as follows. Three mechanisms have been identified for biomolecules to travel from circulation into saliva [5,6]: active transportation for various proteins such as secretory IgA and immunoglobulin E, passive transportation for drugs and steroids, and ultrafiltration for small polar molecules such as creatinine. The basis of our prediction method is that some of the disease-associated proteins in circulation can get into saliva through one of these three mechanisms, hence making it possible for us to identify them in saliva even for diseases of distal organs. 

Two large datasets for salivary proteins are publicly available. One consists of 1,166 proteins and 657 of them are also found in human blood [1]. Another one has approximately 2,000 proteins and 26% of them are also found in blood [6]. We hypothesize that salivary proteins are secreted by the salivary glands either from circulation or in response to the biomolecules that get into the glands from circulation. In this study, we focus on proteins that come from the circulation and leave the prediction work of proteins secreted by salivary glands in response to blood proteins that get into the glands as a future study. 

**Figure 1:**
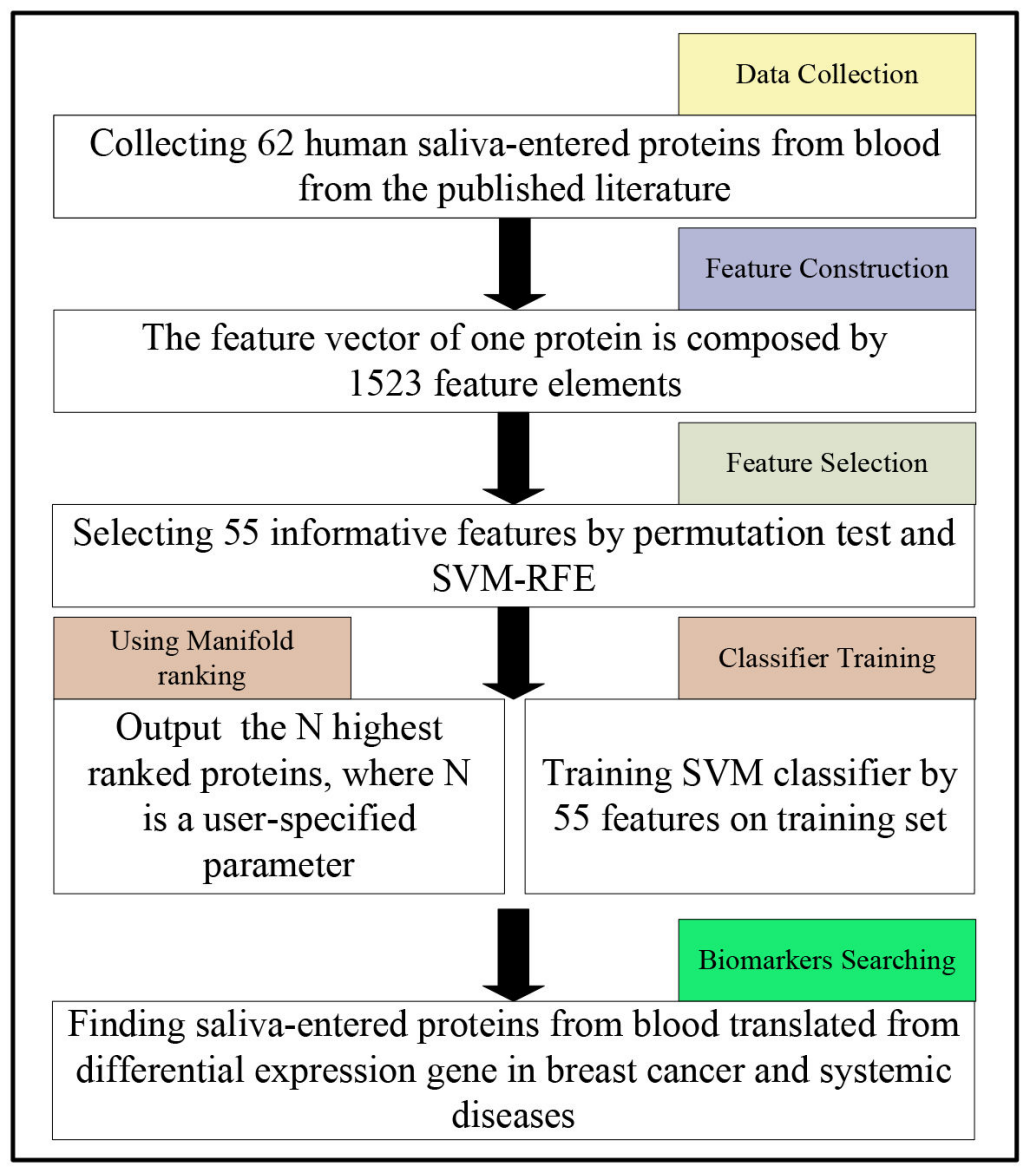
A flowchart of the approach.

We have collected 62 human salivary proteins coming from circulation from the published literature, which have been experimentally detected by multiple salivary proteomic studies, and used them as the initial positive training data. We then expanded this dataset by including additional proteins based on Pfam family information [7]. A total of 261 proteins are selected at the end as the positive training data. We then identified a set of proteins that are deemed not to be able to get into saliva, totaling 6,816, and used them as the negative training data. We then examined a number of sequence and structure-based features to identify those with discerning power between the two sets of proteins. Using these features, we have trained a classifier using a support vector machine (SVM) to predict proteins that can travel to saliva from circulation via salivary glands. In addition, we have also trained a ranking method aiming to rank the known blood-originated salivary proteins the highest among the background proteins, knowing that our negative training data may not be the most reliable. The flowchart of the approach is shown in Figure 1.

We believe that this prediction capability can serve as a general tool for predicting proteins that can travel from circulation to saliva. Hence when applied in conjunction with capabilities for predicting proteins that may be present in circulation of patients of a specific disease, this capability can suggest candidate biomarkers in saliva for that disease. Using this tool along with gene-expression data of breast cancers and a prediction tool for blood-secretory proteins [8], we predicted 31 candidate proteins in breast cancer patients’ saliva. 

## Results

### Features of blood-originated salivary proteins

**Figure 2:**
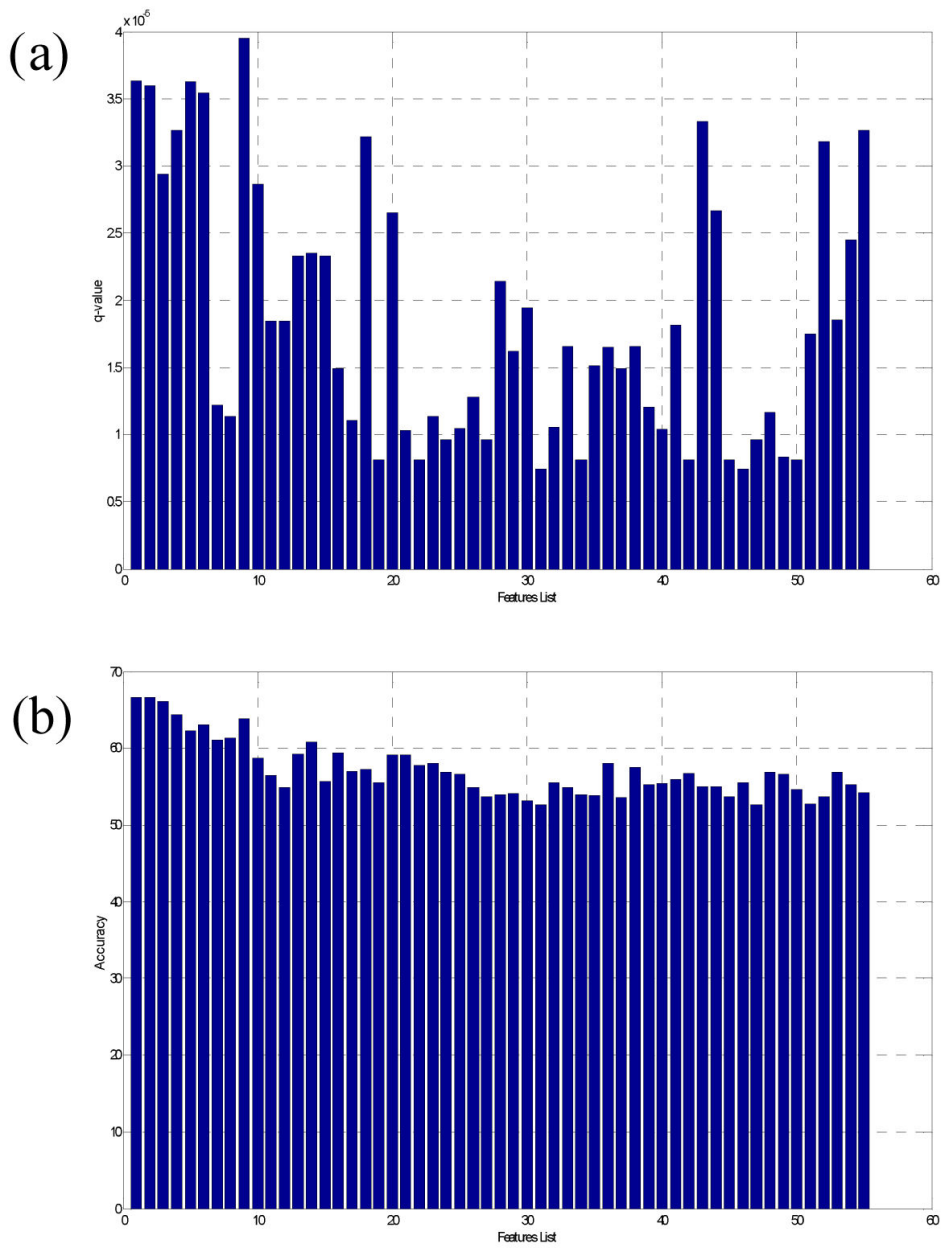
The q-value and accuracy of the 55 selected features.

With the aim of training a SVM-based classifier and rank the predicted proteins, we have examined a total of 34 protein features (see Table S1 and Material and Methods), represented as a feature vector of 1,523 dimension. We then trained a classifier with a linear kernel using these features calculated on proteins in both the positive and negative training sets, aiming to derive a classifier that can best distinguish the positive from the negative samples. We then checked which feature elements are relevant to the final classification performance by using a feature selection procedure, and removed all the irrelevant ones, giving rise to 55 final feature elements. Then a manifold ranking method [8] is trained based on the selected feature elements with the performance given in Table S2. We have assessed the contributions by the 55 feature elements to the classification accuracy, using a statistical significance q-value [9], and found that the q-values for the 55 feature elements are less than 4.0E-5, as shown in Figure 2(A). We have also compared the classification performance based on the 55 features *versus* the top 10 features, and noted that there is a clear difference in performance, as shown in Figure 2(B). The following features are the most important ones to our classification accuracy, ranked in the decreasing order of their contribution to the classification results: radius, Moran autocorrelation, hydrophobicity, Geary autocorrelation, amino acid composition, normalized Moreau-Broto autocorrelation, dipeptide composition, secondary structure composition and polarity. This observation is consistent with our general understanding of secretory proteins and salivary proteins. For example, the diffusion coefficient is inversely proportional to the molecular radius [10]. 

### Performance of the SVM model

**Figure 3:**
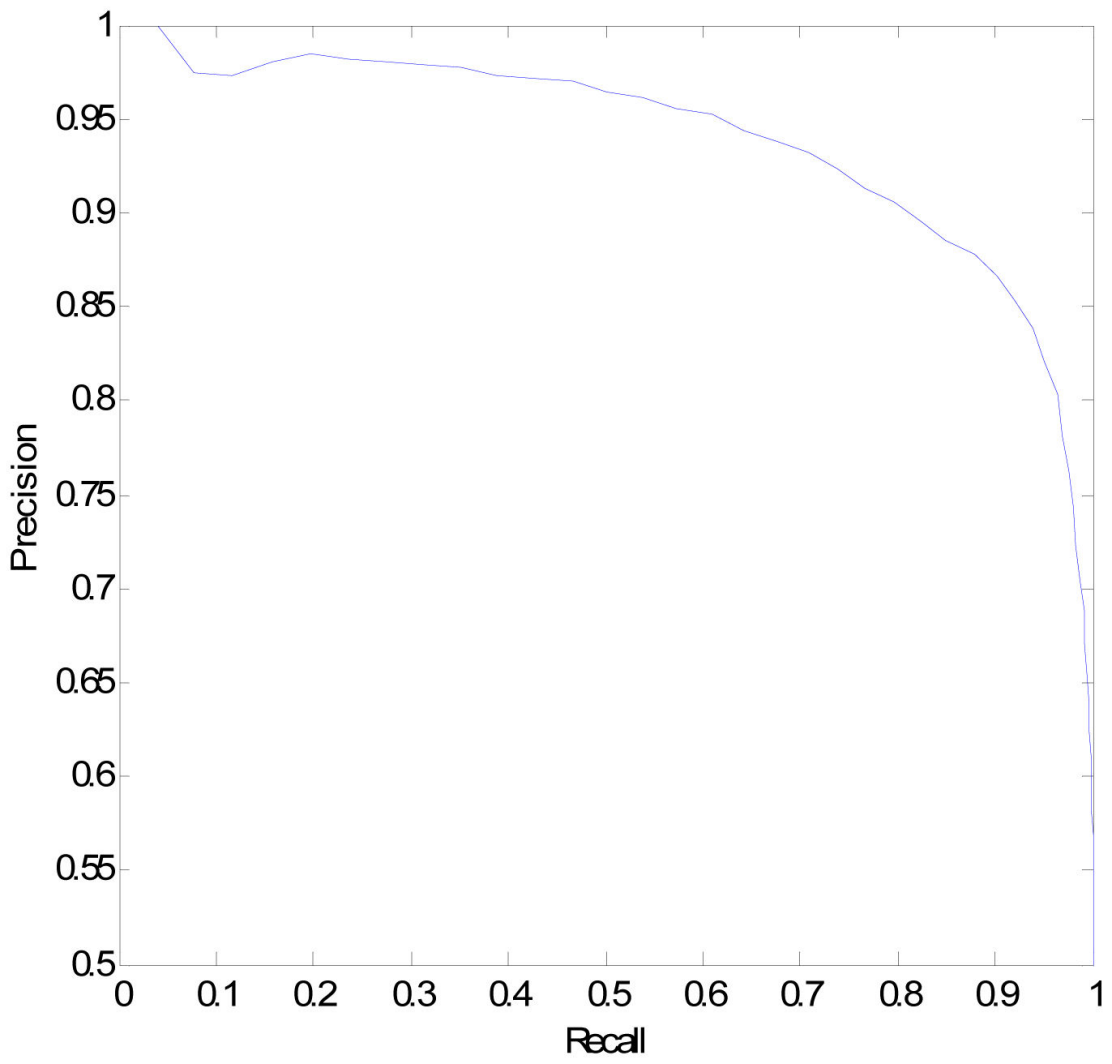
The recall-precision curve.

Based on the 55 selected feature elements, we trained a classifier and evaluated the performance using 10-fold cross validation by repeating the prediction 100 times to derive a performance distribution of the classifier. On the training data, the classifier achieved an average recall and precision at 88.56% and 90.76%, respectively. We applied the general recall-precision curve shown in Figure 3 to the training data with 10-fold cross validation to examine the prediction precision at each recall level. The AUC of the recall-precision curve is 80.96%.

We also used 41 of the 62 collected proteins as the training data that have been reported in the literature before 2000. Out of the 62 collected proteins, 21 are used as the testing data, which have been reported after 2000. On these 21 salivary proteins, our model predicted 14 (76.19%) to be salivary proteins from blood.

### Predicting and ranking the known salivary proteins

We have run the trained classifier on all 20,209 human proteins in the UniProt database [11], among which 5,456 are annotated as secretory proteins according to the Uniprot, SPD [12] and LOCATE [13] databases, and 1,823 have been detected in saliva from previous experiments [1,5,14]. We predicted 2,498 of the Uniprot proteins as salivary proteins from circulation, accounting for 12.36% of the 20,209 Uniprot proteins. Of the 2,498 proteins, 239 (13.11%) are among the 1,823 proteins that have been previously identified in saliva experimentally. 

We have also ranked the human proteins in UniProt using a manifold ranking method as done in our previous work [8]. 62 known salivary proteins coming from circulation were assessed in terms of their ranking to be salivary proteins. By using the 62 proteins as positive dataset, 27 (43.55%) of the 62 proteins and 136 salivary proteins are ranked among the top 1,000 proteins. By using the expanding 261 proteins as positive dataset, 34 (54.84%) of the 62 proteins are ranked among the top 1,000 proteins. Among these 1,000 proteins, 155 are known to be salivary proteins identified from other sources (see Table S3 for the list of the protein names; and also see Material and Methods). While we do not know if the prediction of the remaining 845 proteins being salivary proteins is correct or not, we suspect that some of them are indeed salivary proteins. For example, protein Endothelin-1 (P05305), ranked the 728th, has been implicated in cancer[15], and could be a good salivary biomarker for OSCC development in oral lichen planus patients [16]. Tissue inhibitor of metalloproteinases 1 (TIMP-1) (P01033), ranked the 434th, has been identified as a potential biomarker in diseases such as cancer, cardiovascular diseases and diabetes. Moreover, this protein has been reported to be a salivary protein [17]. 

After the training of our classifier, we did another round of literature search for additional salivary proteins that have been associated with human diseases and do not overlap with our training data. Overall 47 salivary proteins are found, shown in Table S4. These proteins are relevant to different diseases such as periodontal disease [18,19], oral squamous cell carcinoma [20,21], Sjögren's syndrome [22-24], breast cancer [25-29], malignant pelvic tumors, and malignant ovarian tumors [30]. We found that 3 (6.38%) of these 47 proteins are ranked among the top 1,000, 8 (17.02%) among the top 2,000 and 12 (25.53%) among the top 3,000, as shown in Table 1. The p-values for having such rankings if assuming that the ranking is random are 0.211, 0.088 and 0.038, respectively.

**Table 1:** Comparison of the ranking result with human saliva biomarkers for many sorts of diseases.

Total protein number	Known salivary biomarker number	Top number	Salivary biomarker included in top number	P-vlaue
20209	47	500	1	0.367
20209	47	1000	3	0.211
20209	47	1500	6	0.076
20209	47	2000	8	0.088
20209	47	2500	11	0.026
20209	47	3000	12	0.038
20209	47	3500	13	0.052
20209	47	4000	13	0.123
20209	47	4500	15	0.082
20209	47	5000	16	0.098
20209	47	5500	19	0.017
20209	47	6000	20	0.020

We then carried out a pathway enrichment analysis among the top 1,000 ranked proteins, using DAVID [31] against the Gene Ontology, KEGG [32], BBID [33] and BIOCARTA [34] databases to gain an understanding about the cellular functions and subcellular locations of these predicted salivary proteins, using the whole set of human proteins as the background. We noted that the most significantly enriched biological processes are immune response, antigen processing and presentation, cell adhesion, defense response, response to wounding, and inflammatory response. In addition, the most significantly enriched cellular components are extracellular region, membrane and MHC protein complex which all make biological sense (see Table S5). 

### Application to breast cancer for identification of salivary biomarkers

Based on a public transcriptomic dataset collected on breast cancer and matching control samples (see Materials and Methods), we identified 1,502 consistently differentially expressed genes in breast cancer *versus* control tissue samples. We then used the gene expression data as an approximate protein-expression data here; and applied our trained classifier to these proteins and predicted 248 of them to be blood secretory using a prediction tool for blood secretory proteins that we previously developed [8]. Out of these proteins, we predicted 31 are movable to saliva. Table 2 provides the detailed information of these 31 proteins as candidate salivary biomarkers for breast cancer. 

**Table 2:** Proteins as candidate salivary biomarkers for breast cancer.

Gene symbol	UniProt ID	Manifold ranking	Fold change
F10	P00742	195	0.667
CFD	P00746	227	0.593
TIMP2	P16035	241	0.573
CCL14	Q16627	297	0.595
FBLN1	P23142	324	0.663
FBLN5	Q9UBX5	336	0.542
EFEMP2	O95967	363	0.622
IGF1	P05019	394	0.525
EFEMP1	Q12805	439	0.474
AZGP1	P25311	440	1.563
WISP2	O76076	613	0.581
CLEC3B	P05452	720	0.570
CD93	Q9NPY3	724	0.586
LEPR	P48357	1034	0.638
FABP5	Q01469	1072	0.633
IL6R	P08887	1111	0.551
ALCAM	Q13740	1115	1.764
MCAM	P43121	1119	0.527
CFB	P00751	1148	1.647
PDCD6	O75340	1382	1.562
BCHE	P06276	1416	0.665
DMBT1	Q9UGM3	1531	0.632
CD163	Q86VB7	1539	0.628
NCAM1	P13591	1681	0.640
LTF	P02788	1693	1.583
SRPX	P78539	1959	0.556
FBN1	P35555	2192	0.625
CFH	P08603	2400	0.550
VWF	P04275	2518	0.537
CD99	P14209	2867	0.623
TF	P02787	2907	0.578

As of now, very little data is available regarding salivary proteins that can be indicative of breast cancer. The only data we can get hold of is the salivary proteins considered by Streckfus et al. to be informative for diagnosing breast cancer [27]. Their predicted list consists of 37 proteins given in Table S6. We have compared our prediction of 31 proteins with this list, 4 of the 31 proteins are in their list [27-29], as shown in Table 3, which has a p-value at 2.89e-7. The relatively low level of overlap between the two sets of predictions is not particularly surprising, which is consistent with previously published studies by different groups on blood biomarkers for different cancers. This is possibly caused by the differences in detailed conditions under which the biological samples, i.e., cancer tissues and saliva, are collected, as well as the less-than-perfect prediction methods employed, on top of the overall very challenging nature of the problem. 

**Table 3:** Prediction Proteins used as salivary biomarkers for the detection breast cancer.

Not included in the training positive dataset				
**Accession**	**Protein Name**	**Ratio**	**P-value**	**Blood Secretory**
Q01469	Epidermal fatty acid-binding protein	0.633	0.000257	Yes
P02788	Lactotransferrin	1.583	0.000244	Yes
**Included in the training positive dataset**				
**Accession**	**Protein Name**	**Ratio**	**P-value**	
P02787	Transferrin	0.578	0.000013	Yes
P25311	Zinc-alpha-2-glycoprotein	1.563	0.000940	Yes

We have also carried out a pathway and subcellular location enrichment analysis similar to that in the above. We noted that the most enriched biological processes by these 31 proteins are response to wounding, acute inflammatory response, cell adhesion, biological adhesion and immune response, which are all known to be involved in the development of or in defense of cancer. Besides, the most enriched cellular locations are extracellular region and cell surface (Table S7). The most enriched pathways are complement and coagulation cascades, and the second enriched pathways are cell adhesion molecules (CAMs) (Table S8). 

## Discussion and Conclusion

A reliable prediction capability for proteins that can travel from circulation to saliva will represent a highly useful tool as it can provide a candidate list of biomarkers specific to a particular disease. This will allow targeted searches for effective biomarkers in saliva using antibody-based techniques, in comparison with the traditional search strategies by direct comparisons among proteomic data collected from saliva samples of multiple patients and healthy controls, which have proved to be ineffective in searches for biomarkers in blood [8,35] and urine [36]. Here we demonstrated that it is possible to develop one such tool, which by no means represents the possibly most reliable tool for such a prediction. The key contribution of work is the proof of principle that we can possibly identify distinguishing features between proteins that can move to saliva from circulation and proteins that cannot get into saliva. In addition the identified features can also provide useful information to the mechanism studies of how proteins move between blood and saliva. In the future study, we hope that our method could be used in conjunction with the technology platforms for saliva diagnostics, and identify the definitive disease-associated salivary biomarkers.

## Materials and Methods

### Collecting salivary proteins coming from blood and generating negative training data

There is no existing dataset about proteins that can move from circulation to saliva. Proteins that have been found in both salivary proteome and blood proteome cannot serve this purpose since some of the salivary proteins may not come from circulation, instead are secreted from the salivary glands in response to other biomolecules that get into the glands from circulation. Therefore, we collected proteins that can move from circulation to saliva and have been experimentally validated and reported in the literature, such as IgA [6,37], albumin and Zn-alpha2-glycoprotein [38]. 62 such proteins are found from the literature and used as the positive training data, shown in Table S9. Considering the relatively small size of this positive training dataset, we added additional proteins from the same Pfam families of these 62 proteins with sequence similarities lower than 30% to our training set, assuming that proteins in the same Pfam family have the same properties in getting into saliva. To avoid the issue of over-representing any particular family, we limit to have at most five additional members per family, specifically the most distant five members of each of the 62 proteins. This gives rise to a total of 261 proteins, which are used as the positive training data. 

Generating the negative training data is a challenge since our information about which proteins are movable or not is clearly incomplete at this point. We employed a method similar to that proposed by Cui et al. [35] by choosing proteins from the Pfam families not containing any proteins that have been detected in saliva. For each such family, we choose five members as the negative training data. In addition, we keep only those with at least five peptides in the Plasma Proteome Project (PPP) database [39], the largest human plasma protein database. As a result, 6,816 proteins are selected as the negative dataset.

### Feature construction

To train a classifier for proteins that are movable from circulation to saliva, we consider the following features, which can be grouped into four categories: (i) general sequence features such as sequence length, amino acid composition and di-peptide composition; (ii) physicochemical properties such as hydrophobicity, normalized Van der Waals volume, polarity, polarizability, charges, solubility, unfoldability and disordered regions; (iii) domains/motifs such as signal peptides, transmembrane domains and twin-arginine signal peptides motif (TAT); and (iv) structural properties such as secondary structural content and radius of gyration, totaling 34 features, represented by 1,523 feature elements. The details of these features are provided in Table S1. 

### Feature selection and classification

For each protein, we calculated a feature vector of 1,523 dimensions defined above. We first trained a classifier using all the 1,523 feature values on the training data, and then applied a two-stage feature-selection procedure to remove those irrelevant and redundant features. A permutation test and q-value [9] are used to identify and remove the irrelevant features. 10,000 permutations are generated and used to calculate the statistical significance on the relevance of individual feature elements to the prediction accuracy. Then, we used the approach proposed by Storey and Tibshirani [9] to calculate the q-value, which is used to control the False Discovery Rate (FDR) [40], in terms of the p-value obtained from the permutation test. We used 0.005 as the q-value cutoff to remove less relevant features, giving rise to 1,087 retained feature elements. In the second step, an improved feature selection method (SVM-RFE) that considers dependence relationships among features [41] is applied to rank these features. Then we went through an iterative classification and feature removal procedure to have kept only 55 feature elements, which give essentially the same classification result as using the larger feature set. 

A SVM-based classifier is trained on the training data using the 55 feature elements for each protein, and the output is 1 or -1 representing if the input protein is movable to saliva or not. The following parameters are used to evaluate the prediction performance: recall, precision and the area under curve (AUC) of the recall-precision curve [42], defined as follows:

$$\text{recall}=\;\frac{TP}{TP+FN}$$ (1)

$$\text{precision}=\;\frac{TP}{TP+FP}$$ (2)

where *TP* is the number of true positives, *FP* refers to the number of false positives, and *FN* is the number of false negatives.

### A method for ranking predicted salivary proteins

We have also ranked the predicted salivary proteins using the manifold ranking algorithm as in our previous work [8]. The essence of a manifold ranking algorithm [43,44] can be intuitively explained as follows: the problem is defined on two datasets, a true sample set and a background set. Our goal is to rank the individual members of the background set according to their relevance to the true samples. A weighted graph is used to represent the combined true and the background set, with each sample represented as a node of the graph and each pair of nodes being represented as an edge with a weight defined as the similarity between the two nodes in the feature space. Then an evidence propagation process starts, in which each true sample propagates its presence to its neighboring nodes to increase their relevance to the true sample set, where the increased relevance is valued proportionally to the corresponding edge weight in the graph. An overall relevance score of each node is summed over all the scores propagated to it from all the relevant true samples, by which elements in the background set can be ranked at the end. For our problem, the true sample set is the same as the positive training dataset defined in the previous section, and the background contains all the 20,209 human proteins in UniProt minus the positive set. 

### Identification of genes differentially expressed in breast cancer

The microarray gene expression datasets GSE15852 for 43 paired samples of breast cancer and adjacent normal tissues are downloaded from the GEO database of NCBI [45]. For these samples, we applied t test and fold-change to identify differentially expressed genes in cancer *versus* control samples. The expression fold changes of each gene can be calculated using the following formula: 


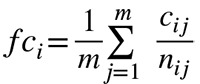
(3)

where *fc*_*i*_ is the ratio of the gene expression value on cancer sample versus control sample of gene i. *c*_*ij*_ is the expression value of gene *i* of cancer sample in patient *j*, and *n*_*ij*_ is the expression value of gene *i* of normal sample in patient j. *m* = 43 is the sample number. The *fc*_*i*_ value is greater than one for up-regulated genes and less than one for down-regulated genes. To identify differentially expressed genes, we choose 1.5 as the threshold of fold change (1/1.5 for down-regulation). Then we can obtain the differentially expressed genes between cancer samples *versus* control samples.

### P-value calculation for comparison of the ranking result with human saliva biomarkers

We calculated the statistical significance p-value assuming the underlying distribution for our problem follows a hypergeometric distribution [46], i.e., the probability of selecting *s* tails in *n* draws without replacement from a finite population of size *N* coins each with an equal probability in selecting a head *versus* a tail containing exactly *S* tails, calculated as follows:

$$P(x=s)=\frac{C\left(S,s\right)\cdot C\left(N-S,n-s\right)}{C\left(N,n\right)}=\frac{\left(\begin{array}{c} S\\ s \end{array}\right)\left(\begin{array}{c} N-S\\ n-s \end{array}\right)}{\left(\begin{array}{c} N\\ n \end{array}\right)}$$ (4)

Where C(a, b)=a!/[b!(a−b)!], *N* is the number of human proteins, *n* is the number of the selected top proteins, *S* is the number of proteins used as salivary biomarkers, and *s* is the number of proteins that are among the 47 known salivary biomarkers and among the top *n* predicted candidate proteins. *N* is 20,209 and *S* is 47. Table 1 shows the p-values, for different *s* and *n*.

## Supporting Information

Table S1 **A list of initial features for prediction of salivary proteins from blood circulation.**
(XLS)

Table S2 **Features of blood-originated salivary proteins as selected by recursive feature elimination method.**
(XLS)

Table S3 **A list of top 1000 blood-originated salivary proteins that ranked by manifold ranking method.**
(XLS)

Table S4 **Salivary proteins that have been associated with human diseases.**
(XLS)

Table S5 **Result of GO enrichment analysis among the top 1,000 ranked proteins.**
(XLS)

Table S6 **A list of candidate up- and down-regulated salivary proteins in breast cancer.**
(XLS)

Table S7 **Result of GO enrichment analysis among the 31 predicted proteins.**
(XLS)

Table S8 **Result of Pathway enrichment analysis among the 31 predicted proteins.**
(XLS)

Table S9 **A list of proteins that can move from circulation to saliva and have been experimentally validated and reported in the literature.**
(XLS)
